# Influence of pycnocline on settling behaviour of non-spherical particle and wake evolution

**DOI:** 10.1038/s41598-020-77682-y

**Published:** 2020-11-26

**Authors:** Magdalena M. Mrokowska

**Affiliations:** Institute of Geophysics, Polish Academy of Sciences, Ks. Janusza 64, 01-452 Warsaw, Poland

**Keywords:** Fluid dynamics, Marine chemistry, Physical oceanography, Limnology

## Abstract

Settling of non-spherical particles in a stratified fluid exhibits complex dynamics in a low-to-moderate inertia regime. Although this process is involved in a wide variety of phenomena in natural fluid systems, its fundamental mechanisms are still unexplored. Understanding of particle settling in microscale is particularly important to explain challenging problems associated with ecological and biogeochemical processes in the ocean due to the delayed settling of particulate matter at pycnoclines. Here, I explore interactions between disk-shaped particles and a stratified fluid with a density transition. By laboratory experiments, I demonstrate that the settling dynamics of the disk crossing a density transition are tightly coupled with the wake structure evolution, and I observe for the first time in a two-layer ambient configuration a bell-shaped structure that forms on a jet after the wake has detached from the particle. Furthermore, I identify hydrodynamic conditions for the variations of settling velocity and particle orientation instabilities. These findings shed light on particle settling mechanisms necessary to explain dynamics of marine particles such as plankton, faecal pellets, and microplastics and may improve the estimation methods of sedimentation processes in various areas of earth sciences and engineering.

## Introduction

Particles descending through a density stratified ambient fluid are widespread in the natural environment^1–3^ as well as in engineering processes^4^. Phenomena related to particle settling are particularly significant in natural waters where the vertical variation of temperature and/or salinity induces density gradients in the ocean, estuaries, coastal regions, lakes, and deep hypersaline anoxic basins affecting the dynamics of particles settling in a water column^2,5^. The main effect is that particles such as faecal pellets, plankton, detrital material, minerals, and microplastics decelerate considerably in the regions of sharp density gradients (pycnoclines), which promotes aggregation process and formation of marine snow. Particles composed of organic matter are considered a significant vehicle for carbon, and settling dynamics is particularly important for better understanding of carbon pump mechanisms^6,7^. Moreover, prolonged residence times of particles at density interfaces has large ecological and biochemical implications, inducing the formation of aggregate thin layers^2^, harmful algal blooms^8,9^, the remineralization of organic matter^7^, and excessive production of exopolymers^10^, which affects the rheological properties of seawater^11^ and causes serious local environmental and social problems^12^. Understanding of these macroscale processes is not possible without accounting for basic microscale settling dynamics of single particles.

Experimental and numerical research has to some extent explained the mechanisms of particle deceleration triggered by a density gradient. Stratification has been acknowledged as a source of additional drag, and several mechanisms for particle deceleration in a stratified fluid have been identified: (1) reduced buoyancy due to the entrainment of lighter fluid from the upper layers of liquid^13–15^; (2) a vertical jet, i.e. the upward motion of an entrained lighter fluid at moderate to high Reynolds numbers (Re) reducing the pressure at the rear of a particle^16,17^; (3) the compression of isopycnals by a settling particle and their restoration to the position of neutral buoyancy^15,18^; (4) the diffusion-limited retention characteristic of porous particles such as marine snow^2,19,20^; (5) internal waves generated by a particle settling in moderate to high Re number^13,15,21^; (6) the flow structure around a particle as a main contribution to drag, which has been demonstrated in a recent numerical investigation questioning the fact that the buoyancy force is triggered by entrained lighter fluid^22^.

A large group of experimental studies on particle settling in stratified ambient fluid has been motivated by a potential application to plankton and marine snow dynamics in pycnoclines present in the ocean. These studies have used experimental configuration with homogeneous upper and lower layers with density transition between them referring to a pycnocline in natural waters^7,13,20,23–25^. Although the range of fluid densities in two-layered experiments reported in the literature usually corresponds to that occurring in aquatic systems, experimental conditions represent larger density gradients than encountered in the ocean^23^. Buoyancy frequency, *N*, expressing stratification strength (Eq. 5), exceeds 1 s^−1^ in mentioned experimental studies, however, such strong stratification is rare in the ocean where transition layers with *N* larger than 0.016 s^−1^ are considered sharp and have been observed to cause accumulation of marine snow^2^. In the same study, buoyancy frequency about 0.039 s^−1^ with thickness of density transition of about 9 m has been observed. Laboratory conditions, including the present study, seem to correspond more directly to aquatic systems where the stratification strength is at least one order of magnitude larger than in the ocean. They are coastal regions, where freshwater overlays seawater; fiords with maximum *N* reaching 0.3 s^−1^ ^26^; hypersaline lakes, e.g. the Dead Sea with *N* up to 0.17 s^−1^ ^27^. However, the most distinct examples are highly stratified systems: deep hypersaline anoxic basins (DHABs) such as Gulf of Mexico (the Orca Basin)^5^, brine pools in the Mediterranean Sea, the Black Sea and the Red Sea^28^, and salt lakes^29^. The increase of salinity by several dozen (psu) over one meter depth has been reported in the Orca Basin^5^ and a sharp halocline has been reported in Ursu Lake (Romania) with salinity difference between homogeneous upper and lower layers about 300 (psu) over 1 m^29^.

The majority of laboratory and numerical studies have considered spherical particles to elucidate settling mechanisms in stratified systems^4,30^, while a large group of marine particles is far from the spherical shape; e.g., marine snow^31^, microorganisms^32^, faecal pellets^33^, bioclastic particles such as shells^34^, and microplastics^35^. Nonetheless, the studies on dynamics of non-spherical particles in the presence of background stratification are underrepresented in literature and their potential effects on micro- and macroscale processes in the environment are still unclear. Existing studies^25,36–39^ have demonstrated that settling dynamics of non-spherical particles in a low and moderate Re number regimes are highly affected by the background stratification, not only in terms of settling velocity but also reorientation patterns. Thus, orientation-dependent settling dynamics of non-spherical particles may have significant effects on processes involving marine particles. A recent literature report has revealed that the pairing of diatoms is orientation-dependent and occurs when organisms are in vertical positions^40^. Therefore it seems to be relevant to consider the orientation of particles as another significant parameter in the settling process through pycnoclines.

Studies on spheres and amorphous aggregates have provided physical explanation for the prolonged residence times of particles in density transitions^13,20,23^. Nonetheless, these findings have not been yet incorporated to widely used ocean sedimentation and biogeochemical models, which may be partially responsible, beside ignoring some biochemical processes, for unreliable estimation of particulate flux^41–43^. The oversimplifications are in two aspects: (1) physical conditions in a water column and (2) physical characteristics of particles. It has been already demonstrated that standard formulas for particles settling in a homogeneous water column misestimate considerably the settling velocity and the residence time of spheres^24^ and disks^39^, since they do not take into account the fact that density gradient in the column of fluid enhances drag acting on a particle. Moreover, researchers are more and more aware that oversimplifications of marine particles shape and density as well as the application of Stokes’ Law, which is dedicated to the settling of a sphere in the laminar Re number regime, may be the source of unreliable estimation of particulate flux in ocean models^44,45^. Better understanding of the dynamics of variously shaped particles settling in stratified systems is necessary to facilitate development of robust modelling approaches.

A large group of marine particles of significant ecological, environmental and sedimentological role, including diatoms, faecal pellets, microplastics and shell fragments, represents disk-like or plate-like shapes. Hence, our fundamental knowledge on the hydrodynamic behaviour of disk-shaped particles in stratified fluid systems has to be improved to gain better understanding of the transport processes of these particles in the environment.

To discuss the problem of the free fall of a non-spherical particle, let me consider a circular disk which is an oblate spheroid with geometrical anisotropy characterized by the aspect ratio, χ = *d*/*h* (χ = 5, χ = 7, χ = 40, χ = 60), where *h* is thickness of the disk, and *d* is disk diameter. Disks are classified as thick (short cylinders), χ $\le$ 10, and thin, χ > 10.

A disk which settles in a homogeneous layer in an intermediate Reynolds number regime, i.e., Re between 1 and 100, where Re = *Ud*/ν, *U*—settling velocity (m s^−1^), ν—kinematic viscosity (m^2^ s^−1^), assumes broadside-on position and steady settling velocity as the effect of pressure distribution around the particle, controlled by inertia^46^. However, as we know from scarce studies performed so far, a non-spherical particle may reorient when it encounters a “sufficiently” strong density gradient^25,30,36,37^. When different buoyancy acts at the opposite edges of a particle, a buoyancy-induced torque appears, which competes with the pressure-induced torque due to inertial effects, and the particle reorients when the buoyancy-induced torque overcomes the inertial one^30^.

Settling dynamics of particles are coupled with the flow field and wake structure formed upstream, which has been well documented for a sphere settling in low and moderate Re number regimes in a linearly stratified fluid. A toroidal standing eddy exists behind a sphere settling in a weak stratification at moderate Re number and the vortex collapses when stratification strengthens and a jet is formed instead^4,16,17^. Moreover, a bell-shaped structure has been observed on a jet in a constant distance behind a sphere^17^. It has been confirmed numerically that the structure is formed as an effect of internal waves, and that the distance between the sphere and the structure depends on the wavelength of the waves^47^. Although we may expect similar effects in the case of non-spherical particles, their mechanisms are likely to be more complex due to orientation instabilities. Scarce studies report the evolution of a wake behind disk settling in stratified fluid revealing a jet moving from the centre of the particle to its edge^25,37^.

**Figure 1:**
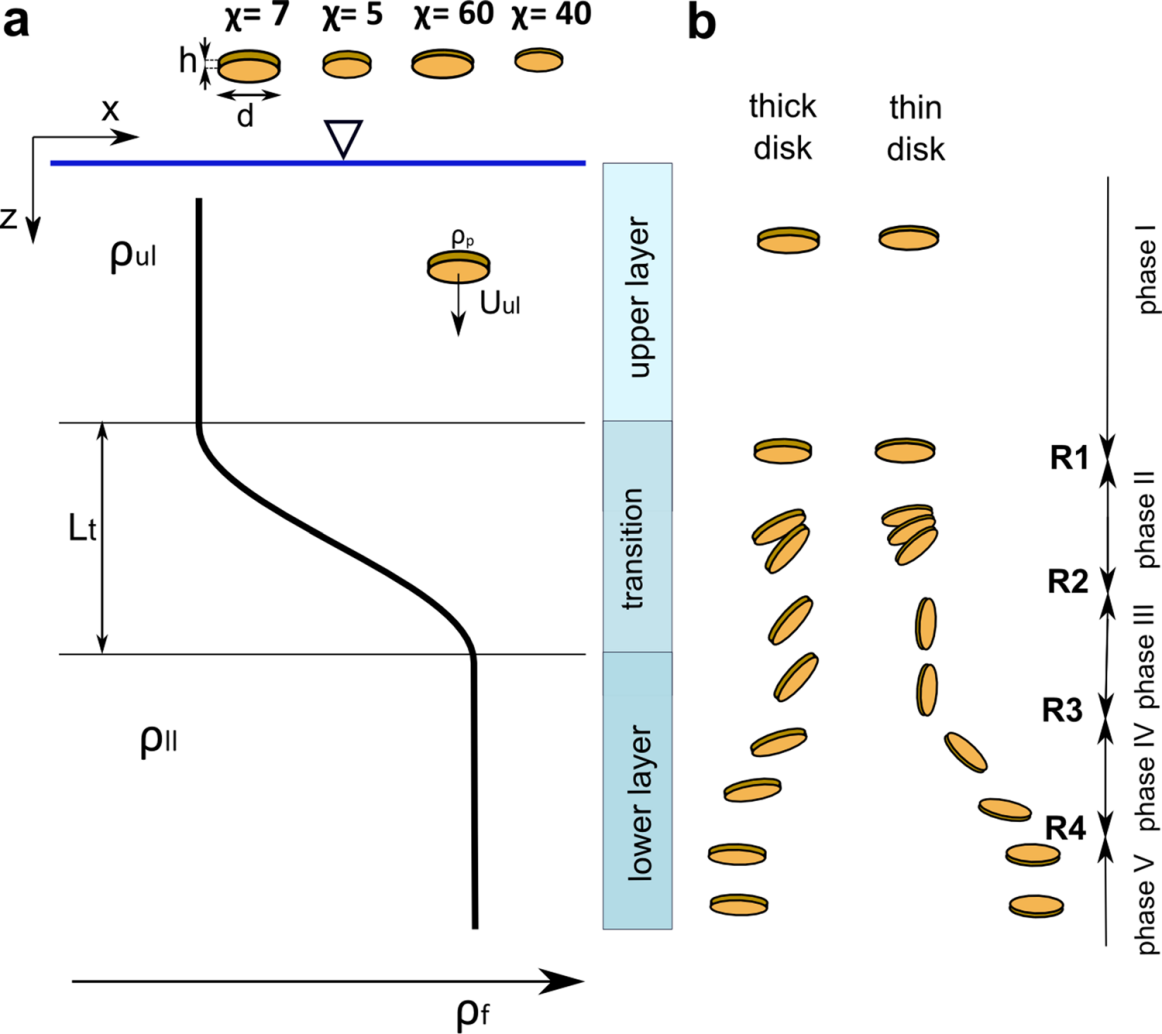
Schematic of stratification conditions in a two-layered liquid column and disk settling dynamics. (**a**) Variation of density with depth in a column composed of an upper layer with density ρ_ul_, a lower layer with density ρ_ll_ and a density transition of thickness *L*_t_. Four types of disks used in the study are shown schematically with aspect ratio χ. A particle of density ρ_p_ settles in the upper layer with terminal velocity *U*_ul_. (**b**) Scheme shows settling dynamics of two groups of disks: thin disks, χ > 10, and thick disks, χ $\le$ 10, with the indication of respective settling phases (I–V) and reorientation points (R1–R4). Differences between settling dynamics of two groups of disks are described in the text.

In this paper I present the results of an experimental study comparing the settling behaviour of thin and thick disks, which may represent various classes of disk-shaped particles in aquatic systems, falling in a stratified salt-water solution. The stratification is two-layered with homogeneous upper and lower layers and a continuous non-linear density transition (Fig. 1a). I provide insight into poorly understood settling behaviour of disk-shaped particles and hydrodynamic interactions between the disk and the wake formed behind the particle in a stratified fluid.

Although the focus of this study is on the mechanics of particle settling in aquatic systems, the results may be of significance to a broad range of environmental sciences including, atmosphere research, geophysics, ecology, and botany considering problems of sedimentation, the hydrodynamics of individual particles, interactions of living and non-living particles with surrounding environment, biogeochemical cycles, the ecology of microorganisms, and to applied sciences including chemical engineering, pharmacy, medicine, metallurgy, mineral processing and petroleum industry where hydrodynamic behaviour of particles is a crucial basic process.

## Results

### Settling behaviour of disks

Disks descended through a stratified water column with variable settling velocity and angular orientation as an effect of fluid density stratification and the geometrical anisotropy of particle. Differences between the settling behaviour of thin disks (χ = 40 and χ = 60) and thick disks (χ = 7 and χ = 5) were observed (see Fig. 1 for reference). Particles were translating through the water column in a low-to-moderate Re number regime with Reynolds entrance number (Eq. 7), Re_ul_, varying between 5 for thin disks and 44 for thick disks, Archimedes number (Eq. 9), Ar_ul_, varying between 3.3 and 16.0, and entrance Froude number (Eq. 11), Fr_ul_, varying between 0.22 and 1.60 (see Supplementary Table 1).

Thin disks followed the settling pattern as described in the previous paper^25^: steady settling in a broadside-on (i.e. horizontal) position in a homogenous upper layer (phase I), reorientation from horizontal to vertical position in the transition layer (phase II), unsteady settling in a vertical position (broadside parallel to the direction of gravity) (phase III), reorientation from vertical to horizontal position (phase IV), and steady horizontal settling in a horizontal position (phase V). The settling velocity reached two local velocity minima—*u*_min1_ on the onset of the first reorientation and *u*_min2_ in phase III. Thick disks showed deviations from this pattern. The main difference is that thick disks do not achieve vertical position, instead assuming tilted position in phase III. Moreover, they do not achieve the second local minimum velocity in the most of cases. All these aspects are analysed below in detail.

**Figure 2:**
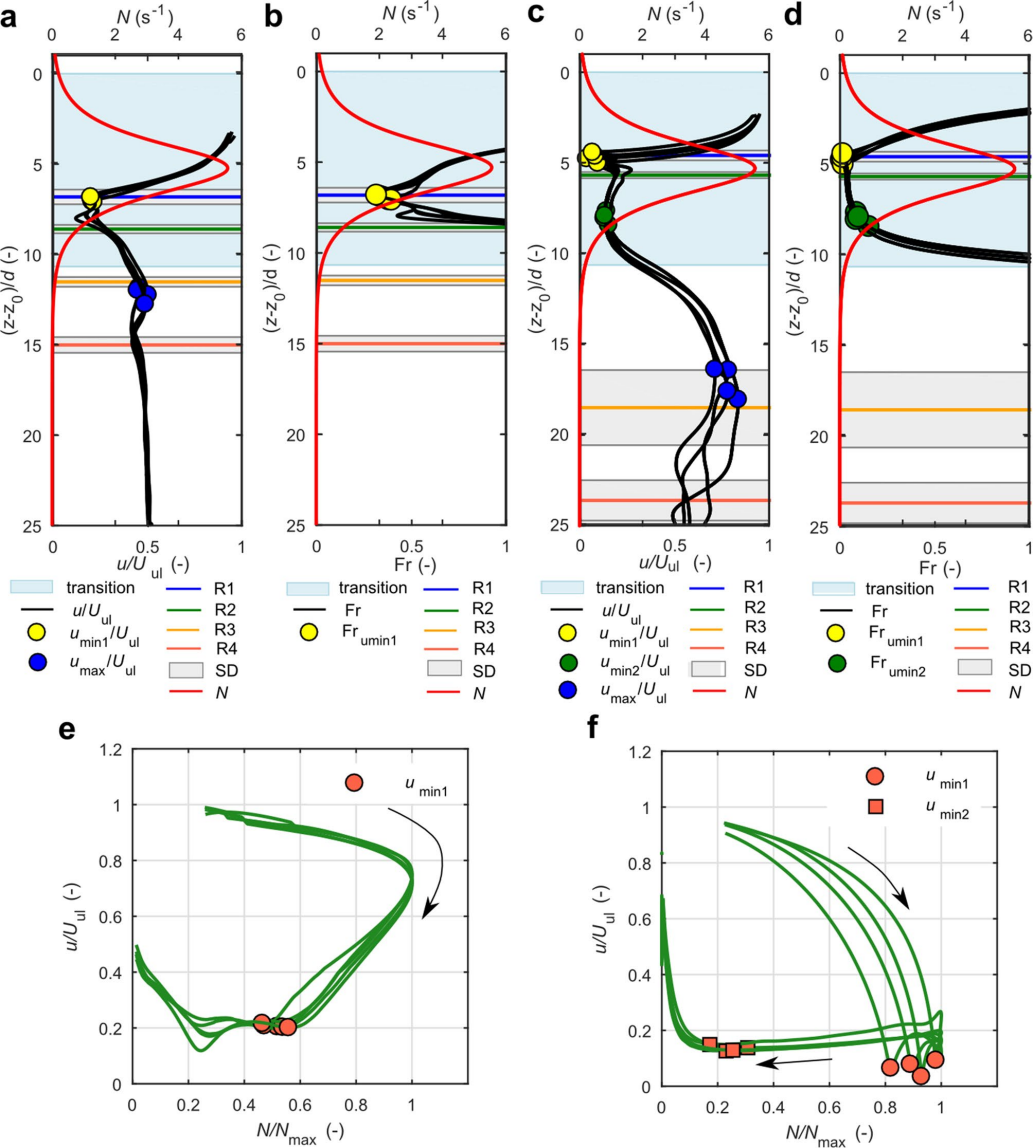
Settling dynamics of disks with different aspect ratios χ = 7 (**a**,**b**,**e**), and χ = 60 (**c**,**d**,**f**) for Set1E1% experiment (see Supplementary Table 1 for details). (**a**,**b**) variation of non-dimensional settling velocity with depth, *z*—vertical coordinate, *z*_0_—location of upper boundary of transition layer, *d*—disk diameter, *u*—instantaneous settling velocity, *U*_ul_—terminal settling velocity in the upper layer, *N*—buoyancy frequency variable with depth, R1, R2, R3, R4—location of reorientations; (**b**,**d**) variation of Froude number with depth; *u*_min1_, *u*_min2_ and *u*_max_ refer to characteristic velocities. (**e**,**f**) variation of settling velocity with buoyancy frequency, an arrow indicates data order from the beginning to the end of settling, *N*_max_—maximum buoyancy frequency.

The impact of particle geometry on settling dynamics was analysed. Figure 2 presents the results for two selected datasets representing thick (χ = 7) and thin (χ = 60) disks for the same stratification conditions (E1%); other results are presented in Supplementary Figs. S4–S6. Figure 2a,c show the variation of settling velocity with depth along with the stratification strength, *N* (Eq. 6), and the vertical locations of reorientations and characteristic velocities. Comparison between settling velocity pattern demonstrates that χ = 60 disk reaches the first local minimum, *u*_min1_, accompanied by the reorientation R1 (from horizontal to vertical position) before the peak of buoyancy frequency, while χ = 7 disk achieves these characteristic points after the particle crosses the region of maximum *N*. It means that thick disks experience orientation instability and minimum velocity in a larger distance from the upper boundary of density transition than thin disks. Analysis showed that the distance is about 0.3 and 0.6 of the thickness of transition layer for thin and thick disks, respectively (Supplementary Fig. S7).

It could be observed from Fig. 2c that a thin disk rotates to the stable vertical position and translates in this position even after leaving the transition. Conversely, a thick disk (Fig. 2a) achieves an inclined position that is not stable, and starts to reorient as soon as stratification effects fade at the lower boundary of the transition layer. In phase III, a thick disk settling in a tilted position keeps on accelerating, while a thin disk decelerates and its velocity reaches the second minimum value.

The analysis of settling behaviour of particles along with parameters characterizing particle motion and stratification conditions provides some insight into the settling process. The Froude number variable with depth, Fr(*z*), is considered to characterize conditions in the density transition (Eq. 12) and the results are presented in Fig. 2b,d. For the purpose of the analysis, results for Fr up to 1 are presented. Reynolds number, moment of inertia, and Archimedes number are also considered to gain insight into the process. Thin disks settle in a moderately low Re number regime in an upper layer with Re_ul_ varying between 5 and 9. The inertia of thin disks decreases considerably in the transition layer above the region of maximum *N*, reaching values Re(*z*) ~ 0.5 for χ = 60 and Re(*z*) ~ 1 for χ = 40 at the onset of the first reorientation R1 were Fr(*z*) achieves the minimum. Consequently, inertial forces are small enough to be overcome by buoyancy forces. Conversely, thick disks translate through an upper homogeneous layer with higher inertia (Re_ul_ in the range between 20 and 44) and the drop of inertia sufficient for reorientation is possible only below the region of maximum *N*, where Reynolds number achieves minimum values (Re(*z*) ~ 6 for χ = 5 and between 6 and 9 for χ = 7). Minimum Re(*z*) is accompanied by the minimum settling velocity *u*_min1_, and minimum Fr(*z*), indicating that the effect of stratification is the strongest compared with the inertial effect of a settling particle.

The fact that a thick disk starts to rotate deeper in the transition layer than a thin disk is explained by the larger moment of inertia of thick disk (dimensionless moment of inertia, I*^46^, is of the order 1 × 10^–3^ for a thin disk and 1 × 10^–2^ for a thick disk). Consequently, larger buoyancy-induced torque is necessary to overcome the inertial one than in the case of thin disk, which is achieved deeper in the density transition.

Archimedes number (Eqs. 9, 10) shows higher values for thick disks than for thin disks with a clear dependence on particle mass—the lighter particle the smaller Ar (see Supplementary Fig. S8). Archimedes number decreases with depth (see Ar_ul_ and Ar_ll_ in Supplementary Fig. S8a,c,e). Data demonstrate that thin disks (χ = 40 and χ = 60) achieve the first minimum local velocity for Archimedes number only slightly lower than the Archimedes number in the upper homogeneous layer, Ar_ul_ (Ar_umin1_/Ar_ul_ from 0.95 to 0.98), while thick disks achieve the minimum velocity for Ar_umin1_/Ar_ul_ varying between 0.55 and 0.70, which is very close to Ar number in the lower layer. This is another indication that thin disks start rotation just after entering the density transition, while convenient conditions for reorientation of thick disks occur at the depth where gravitational forces due to density difference between disk and fluid are close to that in the homogeneous lower layer.

A pronounced difference between Fr(*z*) below the level of the first reorientation, R1, is observed between χ = 60 and χ = 7 disks. While Fr(*z*) increases considerably with depth for a thick disk, nearly constant values are observed for a thin counterpart up to the vertical location where the settling velocity achieves the second local minimum. This is a clear indication that buoyancy effects are dominant over inertial ones for a thin disk within the transition region, and that the settling behaviour of a particle is controlled by stratification. Inertia is small enough (Re(z) between 1 and 2) for thin disks settling in the density transition to let stratification-induced torque dominate and keep the particle in vertical position. Conversely, the ratio of inertial to buoyancy effects is high enough for a thick disk within the transition region to let the particle start R3 reorientation at a higher vertical location, where stratification may still exist. Analysis presented in Supplementary Fig. S7 shows that thick disks start to reorient to horizontal position, R3, at the lower boundary of density transition, while thin disks start to reorient below the density transition, in an average distance 1.4 of the thickness of density transition from the upper boundary of the transition. It should be noted that a larger dispersion of results is observed for thin disks, which is associated with larger sensitivity of thin disks dynamics to the imperfections of particles, i.e. uneven surface and the location of the centre of mass.

The relation between settling velocity and stratification strength (Fig. 2e,f) shows hysteretic character with shapes depending on the geometrical aspect ratio of disks—clockwise for thick disks and 8-shaped for thin disks. It could be observed from the plots (compare with Supplementary Fig. S6) that the first minimum velocity, accompanied with the onset of reorientation, occurs before the peak of *N* for thin disks (*N*/*N*_max_ between 0.5 and 1) and after the *N* peak for thick disks. It could be convenient to use the shape of this relation to differentiate between the buoyancy dominated settling (thin disks) and the settling with pronounced inertial effects within the transition (thick disks).

### Evolution of wake structure for thick disk

Dye visualizations revealed that the evolution of wake structure at the rear of a thick disk (χ = 7) observed in this study is markedly different from the structures reported previously for disks translating through a density stratified fluid^25,37^. This section presents the formation and behaviour of wake, while differences between the observed wake and other forms of wakes are explained in the discussion section.

**Figure 3:**
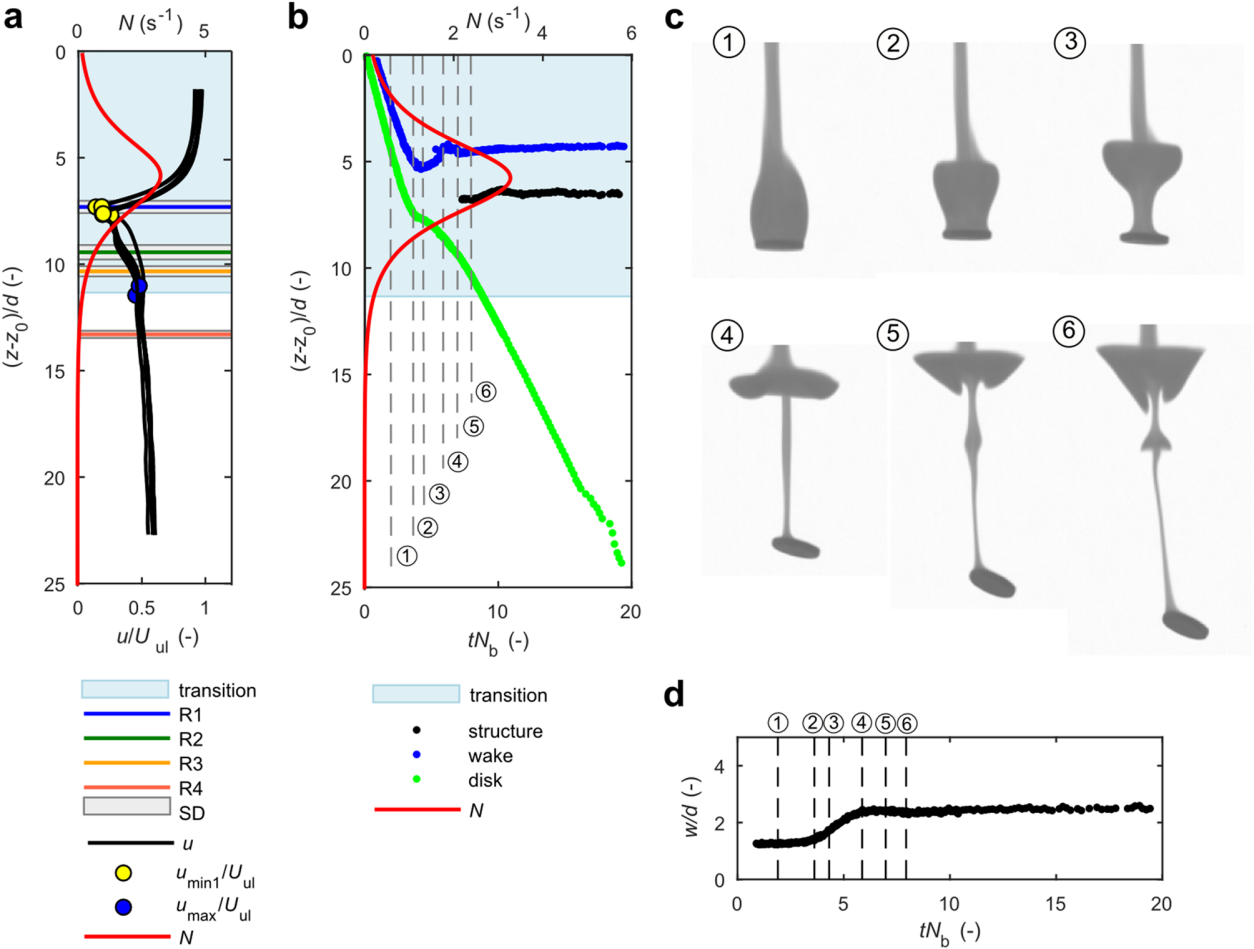
Evolution of wake structure and settling dynamics of a thick disk (χ = 7) for sample data SET3E3% (other results presented in Supplementary Figs. S9, S10). (**a**) Normalized settling velocity of disks as a function of vertical position (*z—z*_0_)/*d*, where *z* = *z*_0_ is the position of upper boundary of density transition. (**b**) Normalized position of the upper edge of a disk (green curve), lower edge of a wake (blue curve) and swelling structure on a jet (black curve) as a function of normalized time, *tN*_b_. Numbers indicate time instants shown in panel (**c**). (**c**) Time sequence of disk translating through the density transition showing selected instants; the instants are indicated in panels (**b**) and (**d**). See Supplementary Movie S3. (**d**) Temporal evolution of wake’s width, *w*. Please refer to the text for details.

Results for a thick disk (χ = 7) are presented in Fig. 3, Supplementary Figs. S9 and S10 and Supplementary Movies S1–S3. A wake that forms in the weakly-stratified upper region of density transition comprises a recirculation zone behind the disk in the form of a toroidal standing eddy and a thread-like part (image 1 in Fig. 3c). When the particle and wake experience increasing stratification, the eddy behind the disk flattens, the wake thins at the base, where it is attached to the disk, and starts detaching (images 2–3 in Fig. 3c).

It could be seen from Fig. 3a,b (vertical position corresponding to image 2), that the beginning of wake detachment occurs when the settling velocity reaches minimum value. The disk is in a stable horizontal position with almost no movement until the major portion of wake separates from the disk (image 2). A column forms between the disk below and a wake plume above during the detachment process. When the column lengthens and thins, its stabilizing effect decreases, buoyancy instability appears between the edges of the disk, and the disk starts to rotate (image 4). The column moves to the disk edge while the disk rotates (images 4–6), a process observed earlier in another study^37^. The column could be considered a jet, similar to studies in linear stratification^17,37^, that is driven by the buoyancy of lighter fluid propagating upwards.

Swelling appears on a jet (Fig. 3c, image 5), which changes its shape into a bell-shaped structure similar to that observed for wakes behind spheres falling in a linear stratification^17^. The swelling appears at the depth where reorientation has started (that is, at the level R1 in Fig. 3a) and is likely to be induced by an internal wave generated by the disk rotational movement when the particle reorients from a stable horizontal position. I explain the appearance of this structure as an effect of upward propagation of a lighter fluid in a jet, which proceeds up to the level of its neutral buoyancy. An internal wave accelerates some portion of a fluid in a jet so that it propagates upwards faster than the rest of the fluid in the jet, which is the explanation given in linear stratification^4,17^.

It could be observed in Fig. 3b that the structure propagates slightly upwards at a time instant *tN*_b_ ~ 9. This might be an effect of internal wave, however, the translation is smaller than the diameter of disk, hence the location of the structure could be considered constant depth, which is (*z*-*z*_0_)/*d* ~ 7 for SET3E3% (Fig. 3b), and ~ 6 and ~ 9 for SET3E2% and SET3E1%, respectively (see Supplementary Figs. S9 and S10). This stable location of bell-shaped structure is unlike in a linear stratification where the structure has been reported to move downwards remaining at a constant distance from a sphere^17^.

It could be seen in Fig. 3b (blue line) that a wake (or a plume after wake detachment) rises to the level of neutral buoyancy after the disk has been separated from the wake (image 4 in corresponding Fig. 3c). This observation confirms that a disk settling with high enough inertia drags an attached fluid to the level of higher ambient density. Afterwards, some of the fluid moves upwards to return to the position of neutral buoyancy. This may also excite an internal wave, beside the rotational motion of disk, affecting the formation of swelling on the jet. Figure 3b shows that swelling was observed for the first time after the plume had already moved upwards, which may suggest coupling between plume motion and swelling appearance.

The wake, and next the plume left behind the disk, widens beginning from the point when the recirculation bubble (image 2 in Fig. 3c) start flattening until the full detachment of wake (image 4 in Fig. 3c), which is quantified in Fig. 3d. Width of the wake is defined as a horizontal width of the minimum bounding rectangle containing the wake.

### Critical parameters of settling dynamics

**Figure 4:**
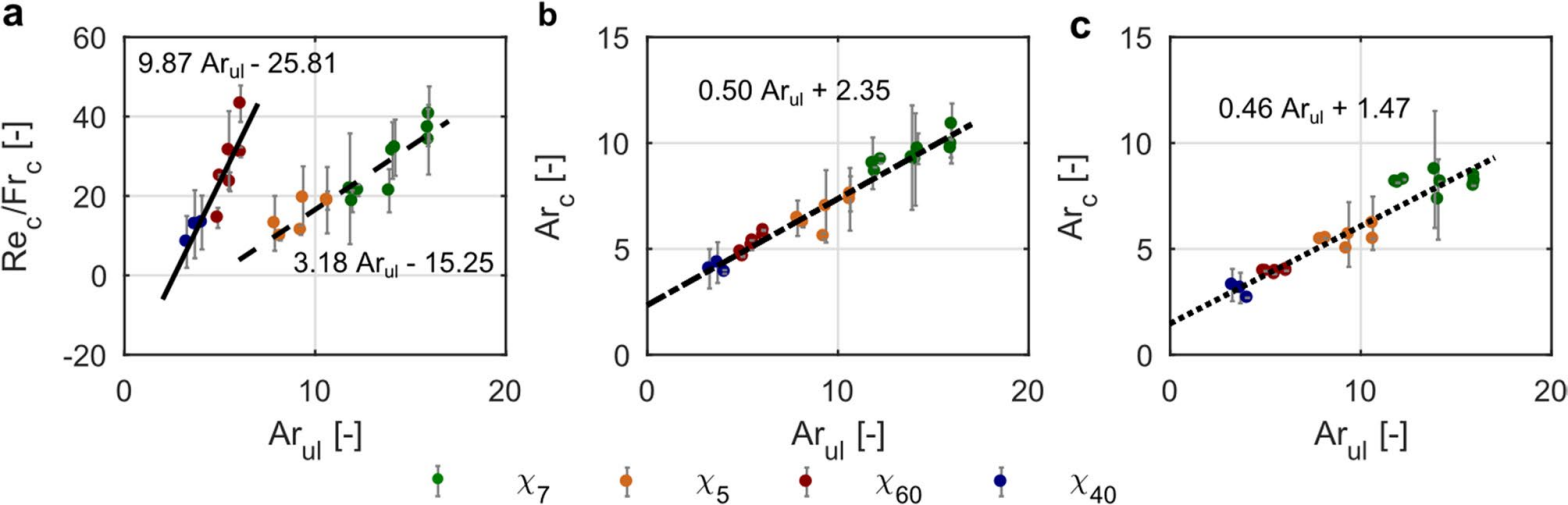
Threshold values for the onset of disk reorientations as a function of the Archimedes number in the upper layer, Ar_ul_, with linear fits. Error bars represent standard deviation and lines represent linear fits. Data from SET1, SET2, SET3 used for analysis. (**a**) Threshold values for the first reorientation, R1, represented by Re_c_/Fr_c_. Two groups formed from thin and thick disks data. (**b**) Threshold values for the onset of the first reorientation, R1, represented by Ar_c_. (**c**) Threshold values for the onset of the second reorientation, R3, represented by Ar_c_.

Data analysis revealed that some critical parameters of disk settling correlate well with the Archimedes number in the upper layer, Ar_ul_ (Eq. 9). Relations between the threshold values for two depth-dependent parameters Ar(*z*) and Re(*z*)/Fr(*z*) in the density transition (Eqs. 10, 13), and, Ar_ul_ were identified (Fig. 4). Threshold values denoted by a subscript “c” were evaluated for the onset of reorientation from horizontal to vertical/tilted position (R1) (Re_c_/Fr_c_, Ar_c_) and for the onset of reorientation from vertical/tilted to horizontal position (R3) (Ar_c_).

Relation between Re_c_/Fr_c_ and Ar_ul_ for thin disks and thick disks followed linear fitting shown in Fig. 4a with the coefficient of determination R^2^ = 0.80 and R^2^ = 0.87 for thin and thick disks, respectively. The analysis of the critical Archimedes number in a density transition revealed a linear relationship between Ar_c_ for two onsets of reorientations (Fig. 4b,c), R1 and R3, versus Ar_ul_ with R^2^ = 0.96 and R^2^ = 0.91 for R1 and R3, respectively.

**Figure 5:**
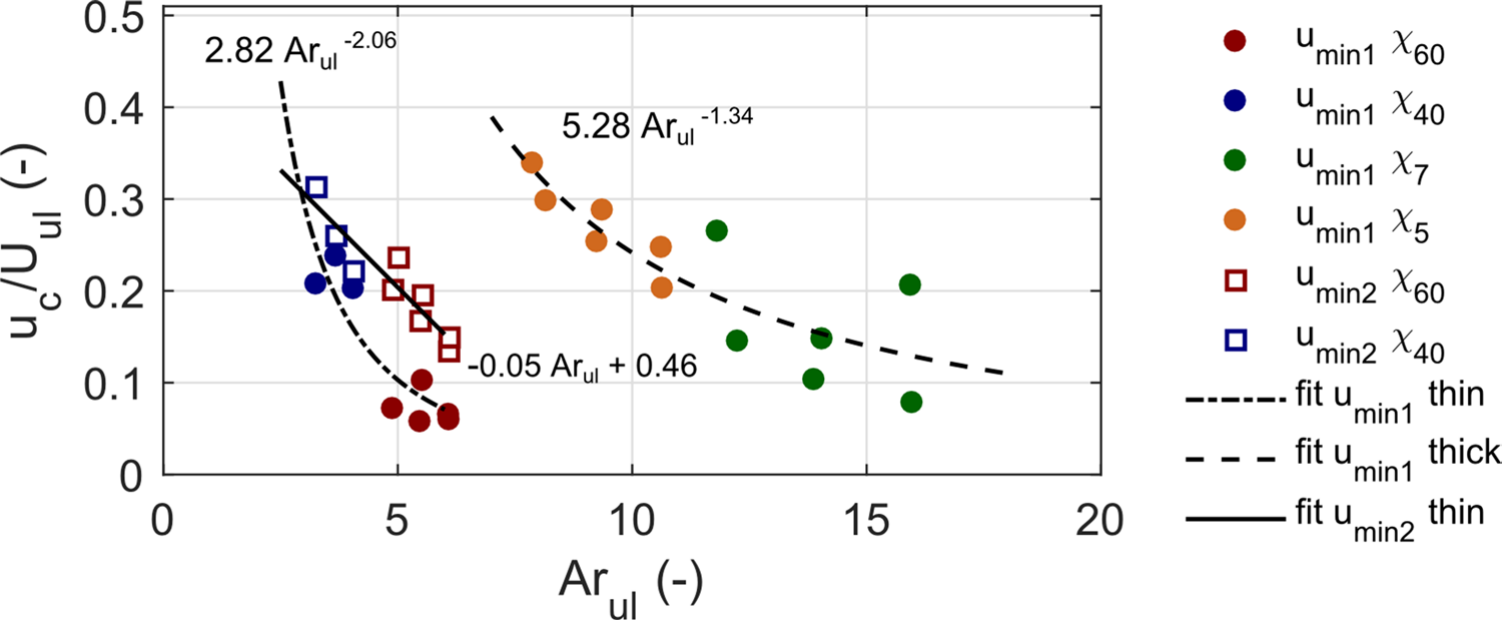
Relations between normalized critical velocities—local minimum velocity, *u*_min1_ and *u*_min2_, and Archimedes number in the upper layer. SET1 and SET2 data are presented. Dashed lines show power-law relationships between *u*_min1_ and Ar_ul_ for thin and thick disks and solid line shows linear fit between *u*_min2_ and Ar_ul_ for thin disks.

The results revealed that disks experienced a significant deceleration as they settled through a density transition. Figure 5 shows the relationships between the non-dimensional first minimum velocity and the Ar number in the upper layer (circles). Results show that the settling velocity values for the first minimum vary between 0.07 and 0.34 of terminal settling velocity in the upper layer. It could be seen from the figure that two groups of data are formed, one for thin and the other one for thick disks. Both of them are well described by power-law functions shown in the figure. In both cases, disks with a larger diameter exhibit more pronounced slowdown. The second minimum velocity for thin disks depends linearly on Ar_ul_ with R^2^ = 0.87.

The results presented in Figs. 4 and 5 show that the critical parameters indicating reorientations and minimum settling velocity depend on the conditions present in an upper layer described by the Archimedes number. Since parameters of settling are not included in the Ar number, this parameter is advantageous in that it could be evaluated based on physical conditions in the column of fluid and properties of particles, and prior information on particle settling dynamics is not required. Thus, presented relations could be used in designing laboratory experiments to induce critical points of disk settling (the onset for reorientations, minimum settling velocity) as well as in numerical studies. Nonetheless, the relations are constrained to the parameter ranges for two-layered fluid configuration considered in this study. Further studies are necessary to apply this type of relations for a wider range of parameters.

### Residence time of disk in transition layer

A residence time, *t*_r_, i.e., the length of time that a disk spends in the transition layer, was evaluated for experimental data. To demonstrate to what extent approximations based on the Stokes formula, widely used in ocean modelling, may misestimate the residence time of disk-shaped particles, I compared the measured residence time of disk, *t*_r_, in the transition layer with the residence time, *t*_r Stokes_, evaluated for an equivalent sphere, that is a sphere of the same density and volume as a disk. The settling velocity of equivalent sphere was calculated using Eq. 1 with depth-dependent fluid density ρ_f_ (*z*):

1 $${U}_{S }(z)= \frac{1}{18}\frac{g{d}^{2}}{\nu }\frac{{\rho }_{p}-{\rho }_{f}(z)}{{\rho }_{f}(z)}$$

where *d*—sphere (or equivalent sphere) diameter (m).

The settling velocity variable with depth *U*_S_ (*z*) was used to evaluate the residence time *t*_r Stokes_. The results are presented in Supplementary Table 2.

The comparison between measured and calculated residence times demonstrates that the approximation based on the Stokes formula underestimates the residence time, which is more pronounced for thin disks. The approximated residence time varies between 0.1 and 0.2 of measured value for thin disks, while it is between 0.2 and 0.5 of measured residence time for thick disks. This comparison indicates that such approximation provides unreliable estimation of the residence times of disks in the transition layer.

Beside the residence time in a transition layer, the length of time spent in a vertical/tilted position, *t*_ph3_, was evaluated as the length of time spent by a disk in phase III to demonstrate a pronounced difference between settling dynamics of thin and thick disks. Moreover, the distance fallen by a disk in phase III, *L*_ph3_, was assessed, since it might be significant for some ecological processes involving living marine particles, as indicated in the introduction.

Non-dimensional parameters, τ_r_ and τ_ph3_, were calculated using terminal settling velocity in the upper layer and the thickness of transition layer to normalize the length of time:

2 $${\tau }_{r} = \frac{{t}_{r }{U}_{ul}}{{L}_{t}}$$

3 $$ \tau_{ph3} = \frac{{t_{ph3 } U_{ul} }}{{L_{t} }}. $$

**Figure 6:**
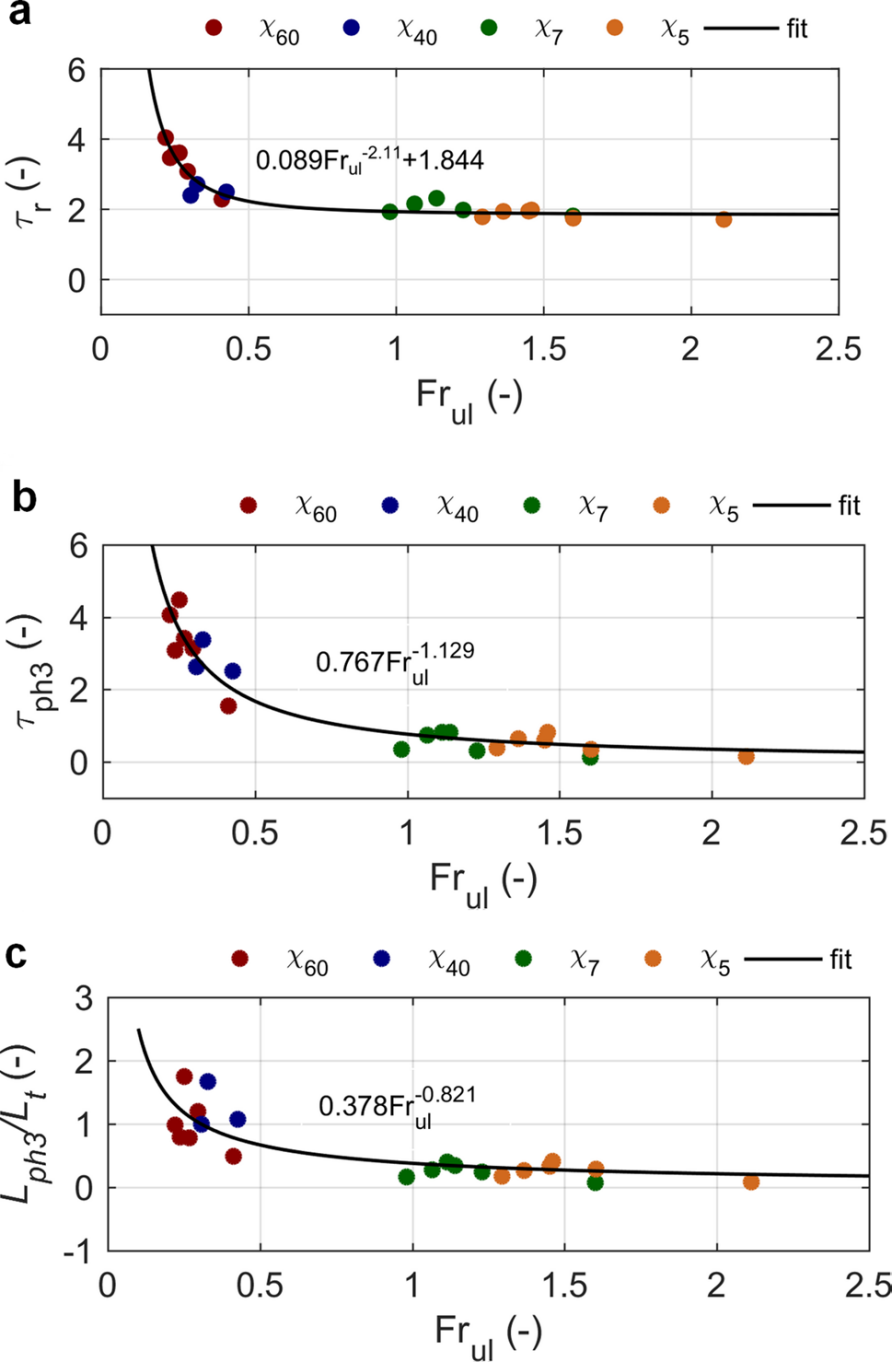
Relations between settling characteristics in phase III and entrance Froude number. SET1 and SET2 data are presented with power-law fits. (**a**) Non-dimensional residence time in transition layer. (**b**) Non-dimensional length of time that disk spends in phase III. (**c**) Non-dimensional distance fallen by a disk in phase III.

Results presented in Fig. 6 reveal that τ_r_, τ_ph3_ and non-dimensional distance, *L*_ph3_/*L*_t_, depend on both stratification conditions and disk dynamics in the upper layer, and power-law relations between these parameters and entrance Fr number, Fr_ul_ (Eq. 11) were found. The relations could be used to assess the dynamics of disks in the transition layer. However, their applicability to the wider range of parameters should be verified in future studies.

## Discussion

I have shown that the settling dynamics of disks descending through a two-layered liquid column with a density transition in a low-to-moderate Re number regime follows complex patterns accompanied by the evolution of wake structure. Comparison between the settling dynamics of thin and thick disks demonstrates that the variation of geometrical aspect ratio by one order of magnitude considerably changes the settling dynamics and wake structure. This may have serious implications on microscale processes involving disk-shaped particles of various dimensions settling in stratified aquatic systems and affect collective motion of particles and interactions between them. Stratification-induced variation of settling velocity and particle reorientations may facilitate the process of marine snow formation enhancing collision between particles representing various dimensions, similarly to the process described for elongated particles^48^. Moreover, the mechanisms of particle reorientation presented herein could help understand ecological processes, such as how diatoms change the orientation to the vertical position, which is crucial in the pairing of these microorganisms^40^. Deceleration mechanisms of non-spherical particles due to density gradients might also facilitate the explanation of increased concentrations of microplastics in a water column below mixed layer in the ocean^49^.

Disks applied in this study could be related to various classes of living an non-living particles in aquatic systems such as diatoms, microplastics, and mineral fragments, since their dimensions (diameters of 2 mm and 3 mm and submillimetre thickness) are characteristic of a large group of naturally-occurring particles. Moreover, considered disk densities are within the range of densities of particles present in natural waters. Particle densities close to that of fluid combined with small disk dimensions induce settling in a low to moderate Re number, which is common in the aquatic environment.

The thickness of density transitions considered in the study is at least one order of magnitude smaller than that observed in nature (from 1 m in hypersaline basins to tens of meters in the ocean) due to constraints related to the size of experimental set-up. Consequently, experimental density gradients are extremely sharp with the buoyancy frequency *N* > 1 s^−1^. Thus, the study could be of particular importance to explain particle accumulation in strongly stratified waters such as hypersaline lakes and large deep hypersaline anoxic basins. Nonetheless, the findings may find application also in real-life cases with weaker stratification, as the results of experimental studies with similar characteristics of density transitions^7,20^.

Timescales of settling process are much longer in natural conditions than considered in the experiments, since thin layers of marine particles may reside in the transition layer over one day^50^. Nonetheless, the experiments provide physical interpretation of delayed settling, that may help understand this process in nature and may facilitate the development of models, for example by taking into account the fact that disk-shaped particles change orientation in response to stratification conditions, and hence settling velocity is modified through orientation-dependent drag. Although the scale of the experimental set-up (spatial and temporal) is much smaller than the scale of real-life sedimentation through pycnoclines, the results demonstrate that it is important to take into account that the settling dynamics of non-spherical particles are readily different from that of spheres. This is particularly important in light of the fact that widely used approximations based on the Stokes formula and equivalent sphere concept provide unreliable estimations of the residence time of disk-shaped particles, as demonstrated in this study.

Wake observations reported in previous studies^25,37^ and the current research allow me to indicate three types of settling behaviour which differ in two aspects: the occurrence of disk reorientation and the form of a wake behind the disk. The analysis of available data suggests that the ratio between inertial and buoyancy forces is a governing parameter for discrimination between the types. When similar stratification conditions are compared, the impact of Re number is also visible, as described below.

Type 1 is represented by a disk settling with a small recirculation bubble in a wake (see Fig. 3b in^25^). No detachment occurs when the disk encounters a density transition, and the wake transforms smoothly into a jet. The disk assumes a stable vertical position and afterwards reorientation. Type 1 has been observed in this study (not recorded) for the Reynolds entrance number (see Eq. 7), Re_ul_ $\in$ (5, 9) and entrance Froude number (Eq. 11), Fr_ul_ $\in$ (0.2, 0.4) and in the previous study^25^ in two-layered stratification conditions for Re_ul_ $\in$ (2, 4) and Fr_ul_ $\in$ (0.2, 0.8) (Fig. 3b in^25^). Similar wake has been observed in a recent experimental study on short cylinders settling in a linearly stratified fluid with *N* = 0.5 s^−1^, Re ≈ 40, and Fr ≈ 0.75^37^, where it was suggested that a jet moving from the centre of the particle to its edge amplifies the torque responsible for cylinder rotation. It should be noted that the stratification strength is constant in a linear, while it varies in a two-layered configuration, hence the comparison between these two cases in not straightforward. The reorientation of a disk and the appearance of a jet was observed in reported studies for low Fr, which may indicate that this type of setting behaviour and wake are characteristic of cases where buoyancy clearly overcomes inertial forces. In such a case, particle translates slowly enough for the wake to adjust to surrounding ambient density, and detachment of lighter fluid occurs gradually from the particle resulting in a jet. Lighter fluid does not penetrate lower denser layer and can gradually adjust to the surrounding fluid. Consequently, no internal waves are generated.

Type 2 is represented by the settling behaviour of thick disks presented in detail in the results section (Fig. 3) and has been observed in this study for Re_ul_ $\in$ (20, 44) and Fr_ul_ $\in$ (1, 1.6). It is characterized by a tilted position in phase III, detachment of a wake and a bell-shaped structure on a jet, which is specific for this type and has not been observed for the other two types described herein. It seems that two conditions should be met for this type of settling behaviour, that is (1) the reorientation of disk and (2) moderate values of Fr number so that a disk is able to drag the wake into the layers of larger density due to inertia high enough compared to buoyancy forces in an upper part of density transition. Buoyancy becomes large enough to induce the reorientation and inertial wave deeper in the transition layer, which affect the wake and the appearance of swelling described in the results section.

Type 3 (Fig. 3c in^25^) is characterized by a stable horizontal position of a disk in the whole density transition. The wake is attached to the disk while elongating as an effect of stratification. The wake detaches uniformly from the whole surface of a disk, and the particle continues to fall in an undisturbed horizontal position, which has been observed for Re_ul_ ~ 100 and Fr_ul _~ 4^25^. In this settling regime, inertia overcomes the buoyancy effects throughout the whole density transition, and a disk does not experience reorientation.

This study provides general classification of wake patterns observed so far. Please note that this rough classification applies to particular physical conditions (kinematic viscosity around 1 × 10^–6^ m^2^ s^−1^, density jumps in the range 0.007–0.045, thickness of transition layer relative to disk diameter 6–46, disk density 1040–1400 kg m^−3^). Nonetheless, it shows the effects that could be expected in aquatic environments where salt is a stratification agent, that is Schmidt number, Sc = 700. Effects observed in this study may be absent when a thermal stratification dominates, since temperature variation results in weaker density gradients and is characterised by larger diffusivity of stratifying agent, and thereby affects settling process to a lesser extent.

The results indicate important mechanisms of particle settling dynamics in stratified systems, including disk reorientations, the variation of settling velocity in a two-layered system as well as metrics for delayed settling (non-dimensional parameters for residence time and time spent by a disk in vertical/tilted position) that could explain settling dynamics of particles in a wider range of conditions through non-dimensional parameters presented in this study. The findings could be useful in the experimental and numerical modelling of settling dynamics of disk-shaped particles in aquatic systems with the focus on the role of disk aspect ratio on sedimentation process. Future studies, experimental and numerical, are necessary to widen the range of experimental conditions including thickness of density transition, stratification strength, and aspect ratio of disks. Moreover, further studies in both linear and non-linear stratification conditions should be performed to study settling of disks in a wider parameter space and to provide thresholds for parameters describing reorientations and wake patterns in these two types of stratified conditions.

## Materials and methods

Experiments were performed in the Hydrodynamic Micromodels Laboratory, Institute of Geophysics, Polish Academy of Sciences, Warsaw, Poland.

### Particles and settling conditions

Four sets of disks ranging in diameter, *d* (*d* = 2 mm and *d* = 3 mm) determined with an accuracy of 0.1 mm, and thickness, *h* (*h* = 50 μm and *h* = 440 μm) determined with an accuracy of 5 μm, have been used in the study. Each set of disks comprised between 12 and 20 pieces. Disks were characterized by a geometrical aspect ratio χ = *d*/*h* and classified into two groups: thin disks—χ = 40 and χ = 60, and thick disks—χ = 5 and χ = 7. Disks were made of acrylonitrile butadiene styrene, manufactured from two types of foils using punchers. The density was ρ_p_ = 1.040 g cm^−3^ and ρ_p_ = 1.050 g cm^−3^ for thick and thin disks, respectively, and was measured by a pycnometer with an accuracy of 0.005 g cm^−3^.

Settling dynamics of disks were measured in a rectangular tank (0.50-m-high, inner dimensions 0.10 m × 0.10 m) made of transparent material (polycarbonate). Disks were settling in a two-layer liquid column composed of aqueous solutions of NaCl. A non-linear density transition formed between an upper homogeneous layer of density ρ_ul_ and a lower homogeneous layer of density ρ_ll_ (see Fig. 1 and Supplementary Fig. S2). The initial interface between the layers during filling the tank was positioned about 0.19 m from the tank bottom so that the bottom wall had no effect on the dynamics of settling disks. The methodology of filling the tank has already been described in^25^. Liquid was sampled in vertical every 5 mm using inspection holes located at a side wall of the tank to measure salinity, followed by the density profile using the methodology described earlier^25^.

Measurements of density vertical variation were fitted to the hyperbolic tangent function (Eq. 4). Next, the buoyancy frequency variable with depth, *N*, was evaluated (Eq. 6). Density transition is considered as part of the liquid column with *N* > 0.2 s^−1^. Since density transition thickness, *L*_t_, exceeds the dimensions of a disk (see *L*_t_/*d* ratio in Supplementary Table 1), the transition is considered continuous.

### Experimental sets

In all experiments, salinity of the lower layer was kept constant (4.6%), while salinity of the upper layer was 1%, 2%, and 3%. Experimental tests are denoted by Ep%χ where p stands for salinity of the upper layer and takes the values 1, 2, 3 and χ is the aspect ratio of a disk 5, 7, 40, 60. Three SETS of tests—SET1, SET2, and SET3 were performed. In each SET, data for three types of ambient conditions Ep% were gathered. Density jump was kept constant for corresponding experiments in different SETS, however, the thickness of transition layer were difficult to be perfectly reproduced (see Supplementary Fig. S2).

An individual particle was released beneath the water surface using a tweezer in the centre of the tank and then the disk settled freely in the stratified water column. The settling of a few disks (2–6) of the same type was observed to assure the repeatability of results. Two-dimensional data on particle settling were collected for all types of disks, for disks χ = 5, 7, 60, and for disks χ = 60 in SET1, SET2, and SET3, respectively (for details refer to Supplementary Table 1). Additionally, dye wake visualizations were captured in SET3. The duration of one experiment was limited to one hour and the number of released disks was limited to several disks to avoid settling-induced and diffusive mixing between layers affecting the density profile. Experiments were carried out in room temperature between 21.4 and 22.9 °C.

### Image acquisition

Two Basler acA2500-60um cameras equipped with Schneider–Kreuznach macro lenses Componon 2.8/28-001 were used. Calibration details can be found in^25^; one image pixel corresponded to 31 μm and a disk was tracked along a distance of 77 mm. The settling process was recorded in SET1 in the upper homogeneous layer and in the density transition using two cameras; one with the top of the field of view positioned about 0.25 m from the water surface and the other with the top of the field of view positioned about 0.21 m from the tank bottom (Supplementary Fig. S1a). Only the second position of the camera was applied for SET2 and SET3 to track disk settling in the transition layer (Supplementary Fig. S1b). Particles were visualized using a shadowgraph method by applying LED panels powered by direct current supply to illuminate a disk from behind the tank. Images of settling disks were recorded with a frequency of up to 60 fps.

### Image pre-processing, processing and settling velocity

Image pre-processing, processing and analysis were carried out using ImageJ and MATLAB® as in the previous studies^25,51^. In each raw grayscale image, a particle was detected using thresholding. The centre of mass, which was next used to evaluate the settling velocity and trajectory, was assessed by identifying the position of the geometric centre of disk projection on a plane. Time-resolved position data were smoothed by the Savitzky–Golay filter applying moving-average cubic polynomial to time-resolved position data (see Supplementary Fig. S3). Next, the central-point difference quotient was used to evaluate settling velocity^25,51^.

### Dye visualizations

Experiments in SET3 were designed to observe the evolution of a wake behind the settling disk. Disk χ = 7 was used in this study. First, settling dynamics of several (5–6) disks were tracked to get repeatable results on particle dynamics (Fig. 3 and Supplementary Figs. S9 and S10). The last disk in experimental series was immersed in a rhodamine B to get the visualization of wake (see Supplementary Movies S1–S3).

### Parameters used in the study

Parameters used in the study are listed below. Please note that some parameters has two forms—single-valued and variable with depth. Parameters variable with depth are used to analyse settling dynamics in the transition layer.

- Hyperbolic tangent function describing vertical density gradient in a liquid column^7^:
  4 $${\rho }_{f}(z)=\left(\frac{{\rho }_{ll}-{\rho }_{ul}}{2}\right)\left(1+\mathrm{tanh}\left(\frac{z-{z}_{0}}{p}\right)\right)+{\rho }_{ul}$$
  where *ρ*_f_—density of fluid (kg m^−3^), *ρ*_ul_—density of homogeneous upper layer (kg m^−3^), *ρ*_ll_—density of homogeneous lower layer (kg m^−3^), *z*—vertical coordinate (m), z_0_, *p*—fitting parameters (m).
- Brunt–Vaisala buoyancy frequency for a two-layered liquid column^14^:
  5 $${N}_{b}=\sqrt{\frac{g\Delta {\rho }_{f}}{{L}_{t}{\rho }_{ll}}}$$
  where *L*_t_—thickness of transition layer (m), *g* = 9.81—acceleration due to gravity (m s^−2^).
- Brunt–Vaisala buoyancy frequency variable with depth^25^:
  6 $$N(z)=\sqrt{\frac{g}{{\rho }_{f}(z)}\frac{\partial {\rho }_{f}(z)}{\partial z}}$$
  where $\frac{\partial {\rho }_{f}(z)}{\partial z}$—background density gradient (–).
- Entrance Reynolds number^24^:
  7 $${Re}_{ul} = \frac{{U}_{ul} d}{\nu }$$
  where *U*_ul_—terminal settling velocity in an upper layer (m s^−1^), *d*—diameter of particle (m), ν—kinematic viscosity (m^2^ s^−1^).
- Reynolds number variable with depth:
  8 $$Re(z) = \frac{u(z) d}{\nu }$$
  where *u*—disk settling velocity (m s ^- 1^).
- Archimedes number for a disk; characteristic length is an equivalent sphere diameter, that is, diameter of a sphere of the same volume^52^:
  9 $${Ar}_{ul} = \frac{d}{\nu }\sqrt{\frac{3}{16}gh\frac{{\rho }_{p}-{\rho }_{f}}{{\rho }_{f}}}$$
  where *ρ*_p_—disk density (kg m^−3^).
- Archimedes number for a disk variable with depth; characteristic length is an equivalent sphere diameter, that is, diameter of a sphere of the same volume^52^:
  10 $$Ar\left(z\right)= \frac{d}{\nu }\sqrt{\frac{3}{16}gh\frac{{\rho }_{p}-{\rho }_{f}\left(z\right)}{{\rho }_{f}\left(z\right)}}.$$
- Entrance Froude number^13,14^:
  11 $${Fr}_{ul} = \frac{{U}_{ul}}{{N}_{b}d}.$$
  where *N*_b_—buoyancy frequency calculated with Eq. 3.
- Froude number variable with depth:
  12 $$Fr(z) = \frac{u(z)}{N(z) d}.$$
- Ratio between Reynolds and Froude number variable with depth:
  13 $$Re(z)/Fr(z) = \frac{N(z){d}^{2}}{\nu }.$$

## Supplementary information

Supplementary Video 1.

Supplementary Video 2.

Supplementary Video 3.

Supplementary Informations.

## Data Availability

Data supporting the findings of this study are available from the author upon request.
