# Supplementary material for: Influence of pycnocline on settling behaviour of non-spherical particle and wake evolution
Source: Sci Rep. 2020 Nov 26;10:20595. doi: 10.1038/s41598-020-77682-y (PMC7692520; doi:10.1038/s41598-020-77682-y)
Supplement: Supplementary file 4 — Supplementary Informations. [file 41598_2020_77682_MOESM4_ESM.pdf]

Supplementary materials

Influence of pycnocline on settling behaviour of non-spherical particle and wake evolution

Magdalena M. Mrokowska

Institute of Geophysics, Polish Academy of Sciences, Ks. Janusza 64, 01-452 Warsaw, Poland
e-mail: m.mrokowska@igf.edu.pl

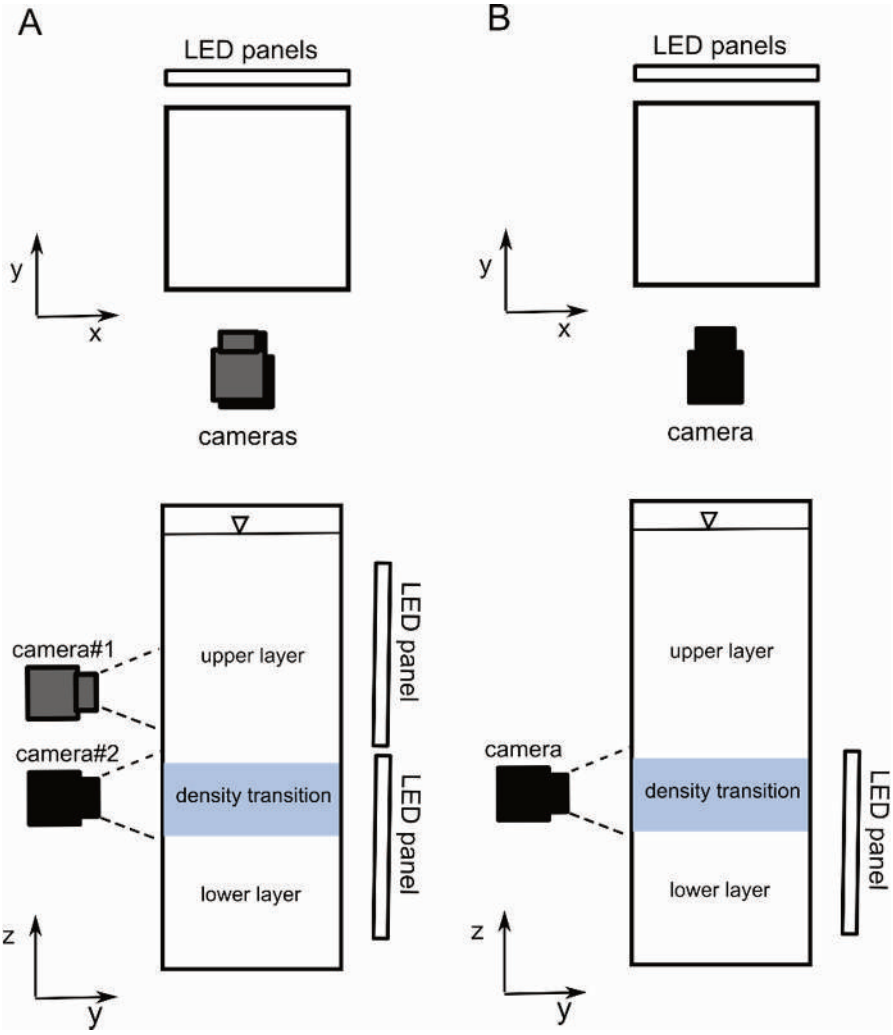

Supplementary Figure S1. Scheme of the experimental set-up a) SET1 experiments with two cameras, upper panel - top view, lower panel - side view, b) SET2 and SET3 experiments with one camera, upper panel - top view, lower panel - side view.

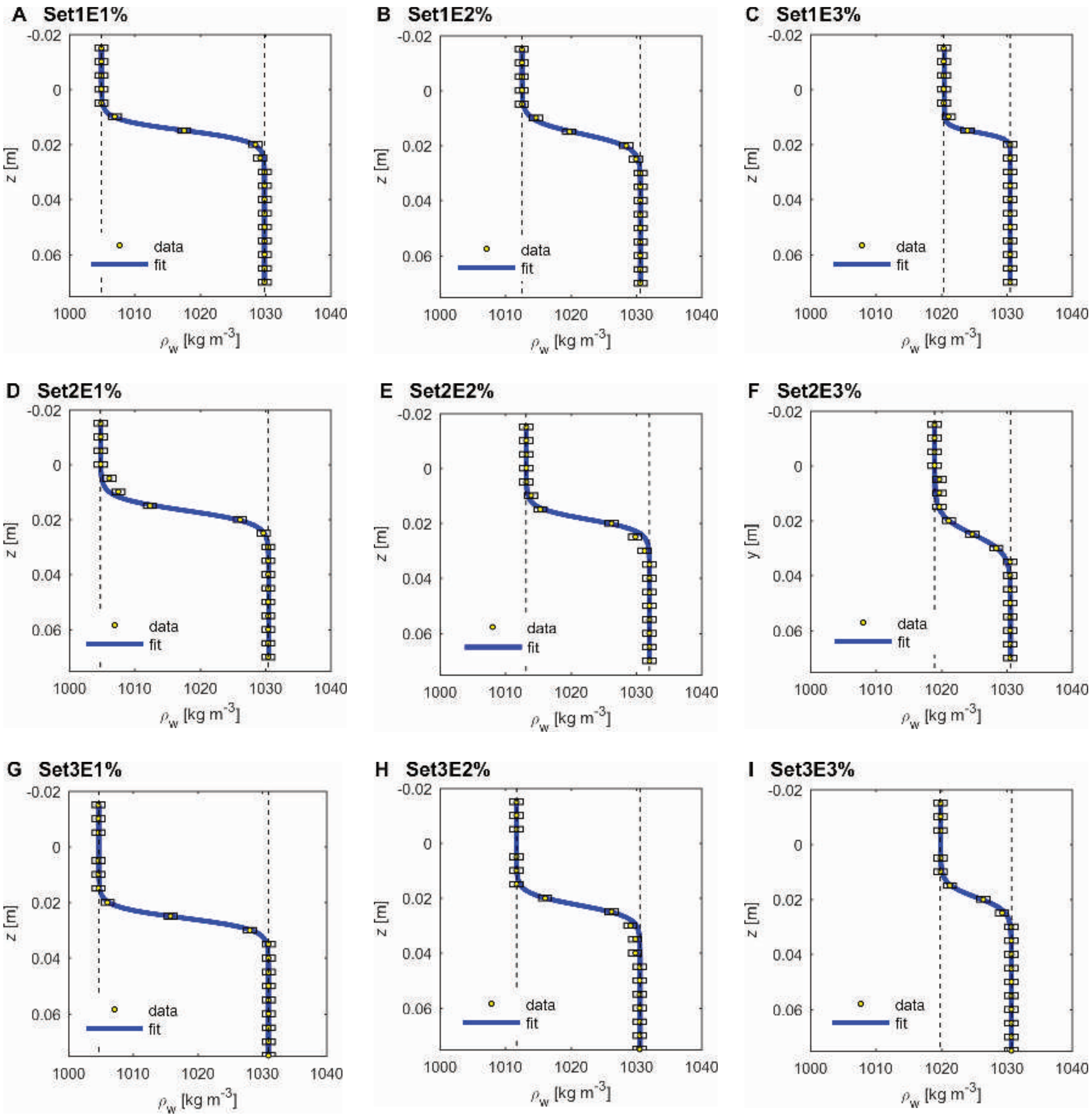

Supplementary Figure S2. Vertical variation of fluid density in the settling tank. Measured density data are shown with error boxes, data were fitted with tangent function given by Eq. (2). $z = 0$ corresponds to the position of density interface set during an experiment.

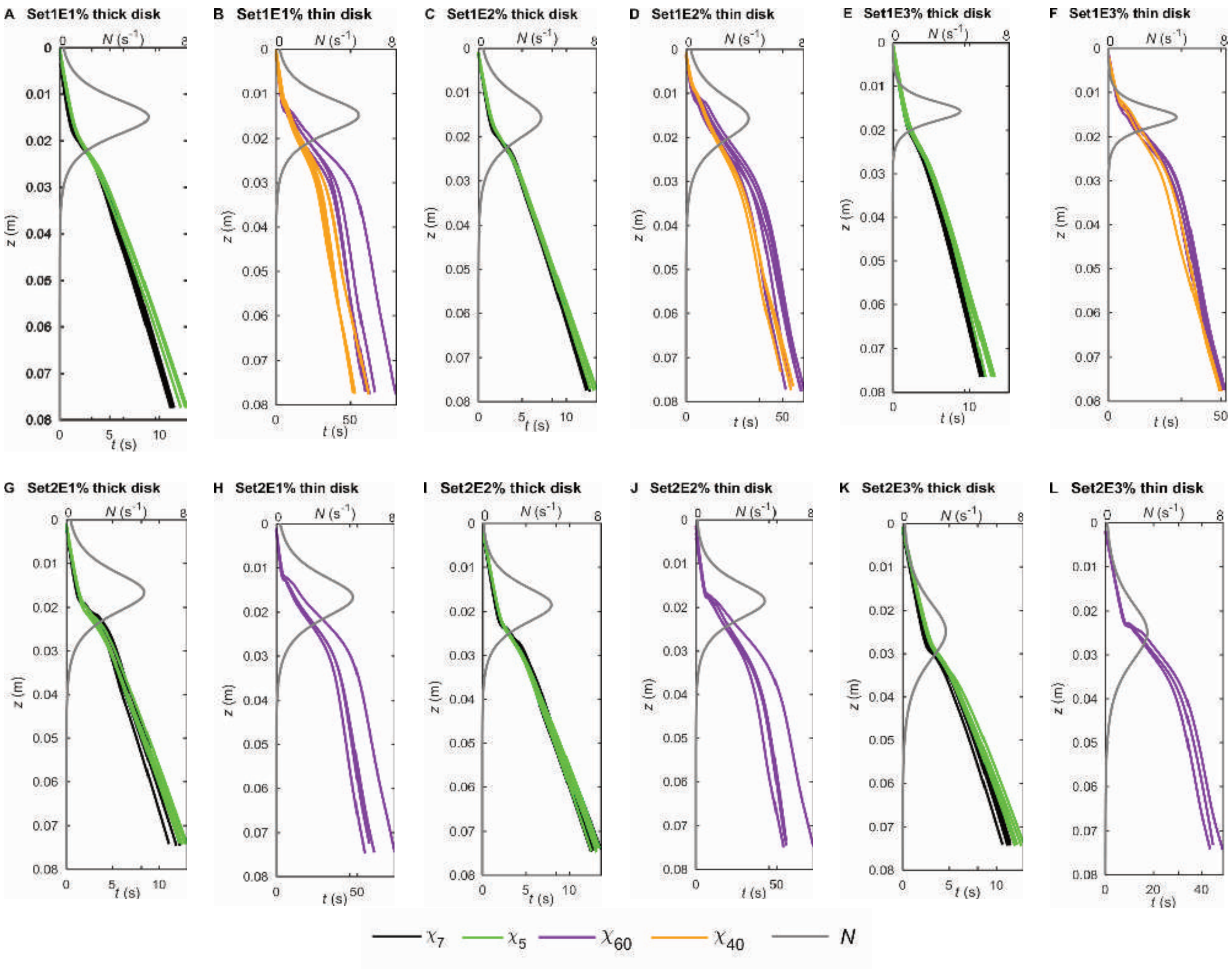

Supplementary Figure S3. Vertical positions of thick and thin disks as a function of time for SET1 (A-F) and SET2 (G-L) experiments. Stratification conditions are shown as buoyancy frequency, *N*. Data for all tests within each experimental set are presented. Data are smoothed using Savitzky-Golay filter and are next used to assess settling velocity (see Methods section).

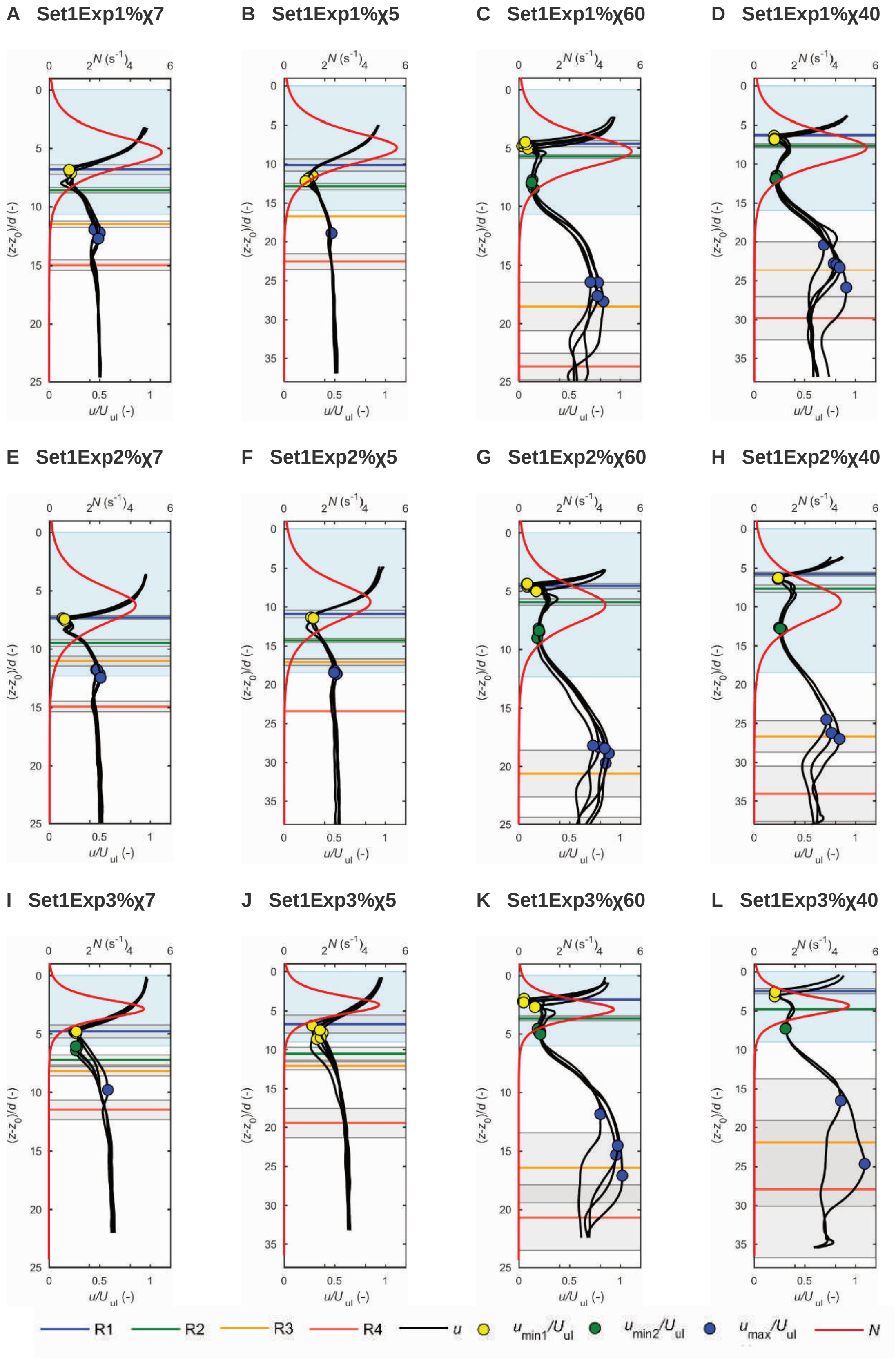

Supplementary Figure S4. Variation of non-dimensional settling velocity with depth, z – vertical coordinate, z_0 – location of upper boundary of transition layer, d – disk diameter, u – instantaneous settling velocity, U_{ul} – terminal settling velocity in the upper layer, N – buoyancy frequency variable with depth, u_{min1}, u_{min2} and u_{max} refer to characteristic velocities and R1, R2, R3, R4 to reorientation points.

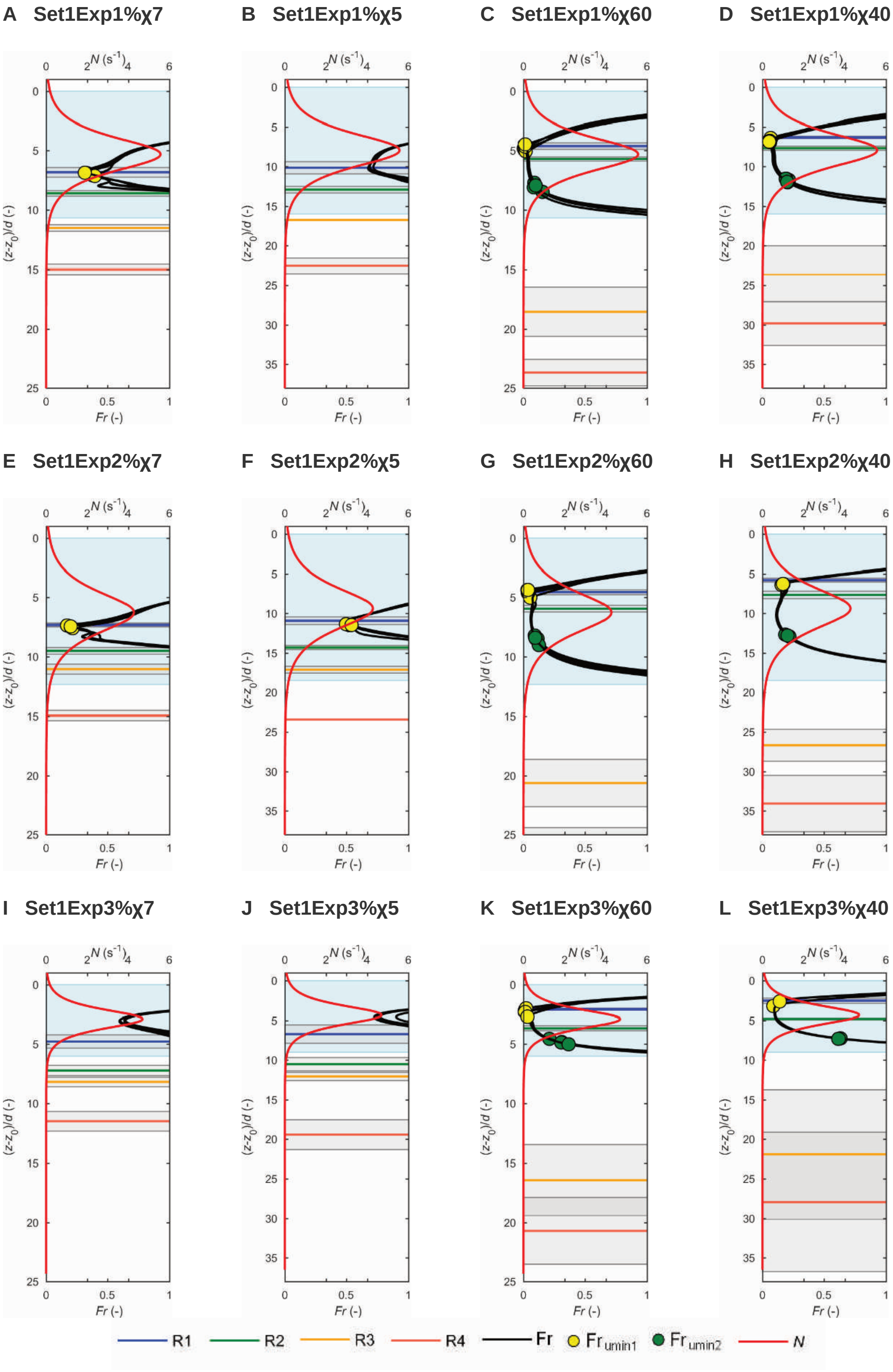

Supplementary Figure S5. Variation of Froude number with depth, z – vertical coordinate, z_0 – location of upper boundary of transition layer, d – disk diameter, u – instantaneous settling velocity, U_{ul} – terminal settling velocity in the upper layer, N – buoyancy frequency variable with depth, u_{min1}, u_{min2} and u_{max} refer to characteristic velocities and R1, R2, R3, R4 to reorientation points.

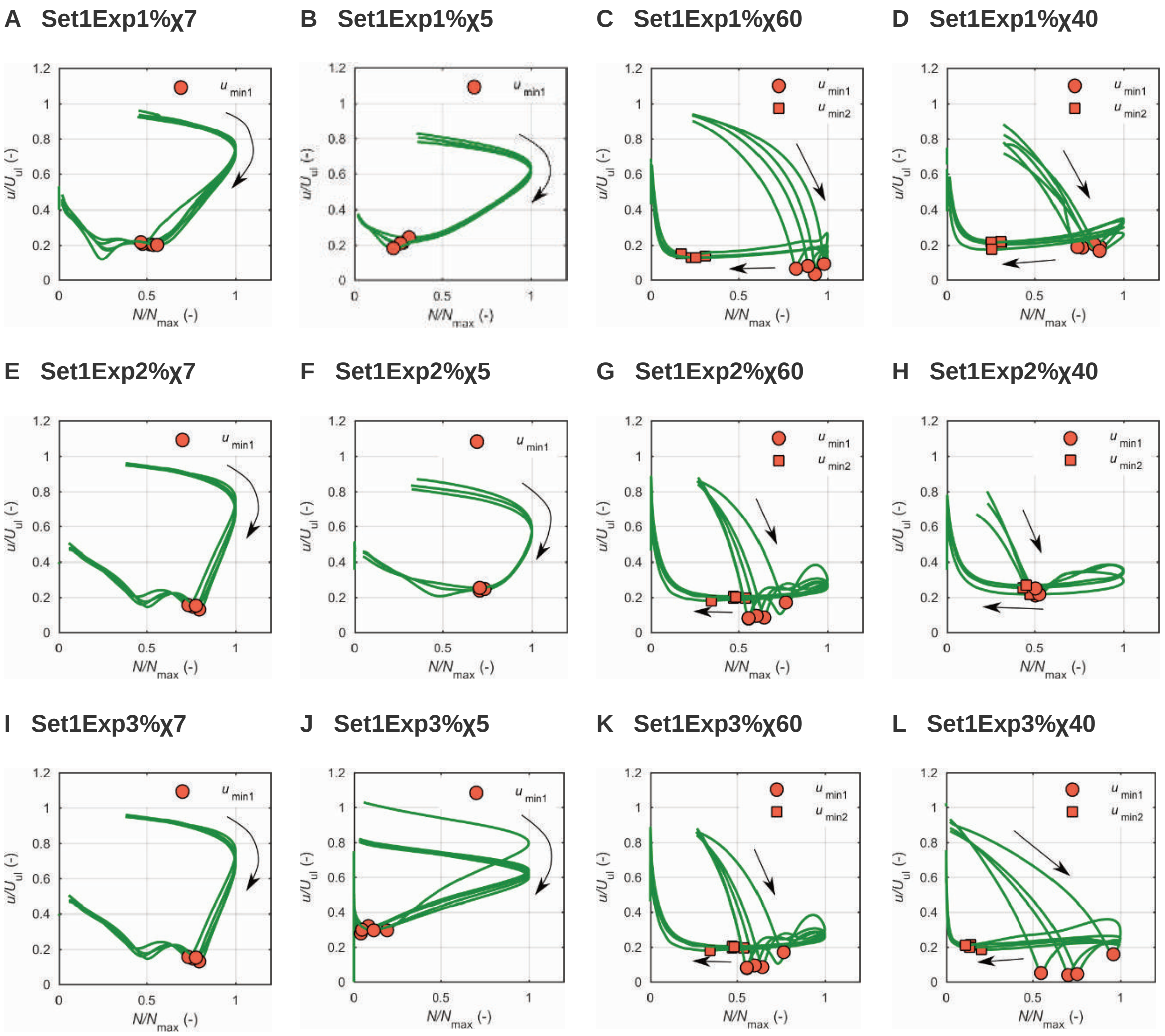

Supplementary Figure S6. Variation of settling velocity with buoyancy frequency, an arrow indicates data order from the beginning to the end of settling, N_{max} – maximum buoyancy frequency.

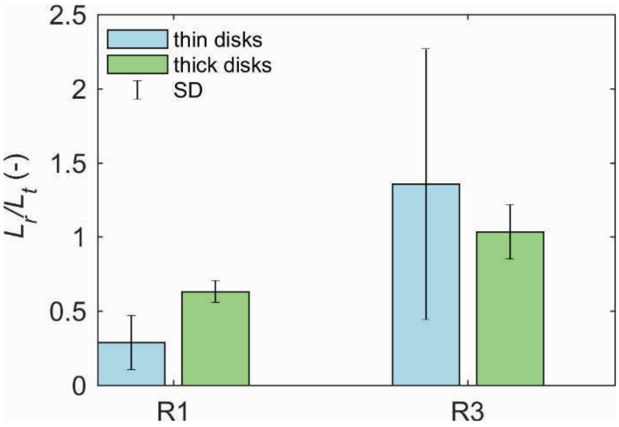

Supplementary Figure S7. Non-dimensional distance between the upper boundary of transition layer and the location of the onset of reorientations R1 and R3 for thin and thick disks. L_r – distance between the upper boundary of transition and the location of reorientation (m), L_t – thickness of density transition (m). Mean values of all data from SET1 and SET2 are presented with standard deviation, SD.

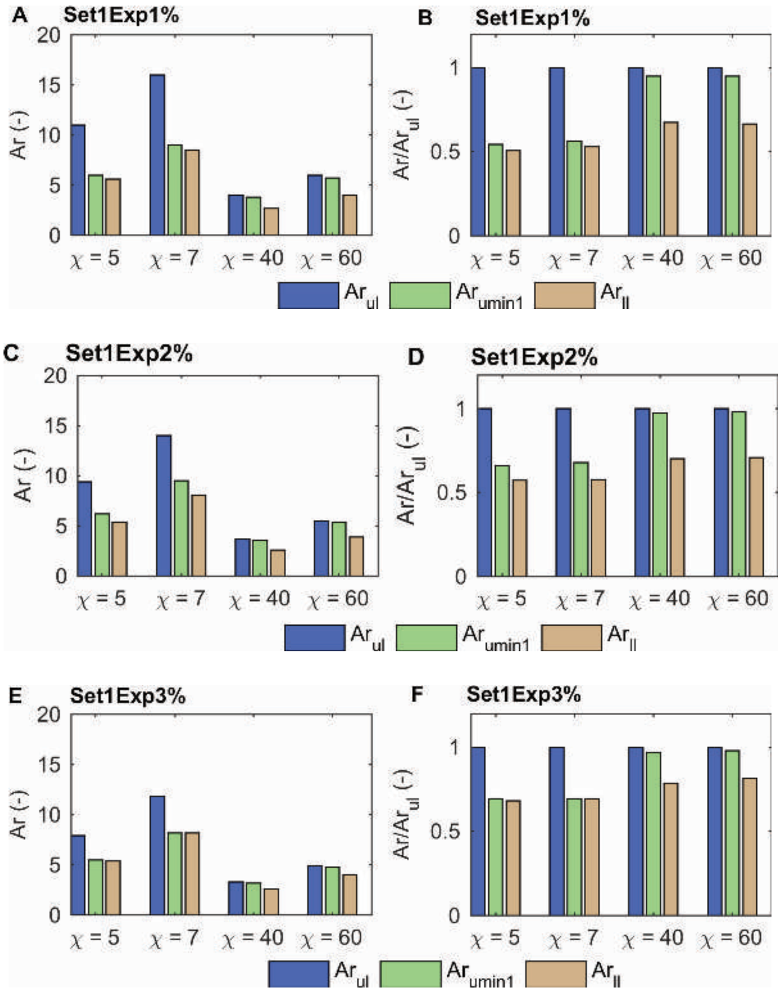

Supplementary Figure S8. Variation of Archimedes number in the column of fluid for disks with various aspect ratios ($\chi = 5$, $\chi = 7$, $\chi = 40$, $\chi = 60$) in three experiments. A, C, E– values of Archimedes number achieved in an upper layer (Ar_{ul}), lower layer (Ar_{ll}) and for the local velocity minimum, u_{min1}, in the transition layer (Ar_{umin1}); B, D, F – values of Archimedes numbers related to Ar_{ul}.

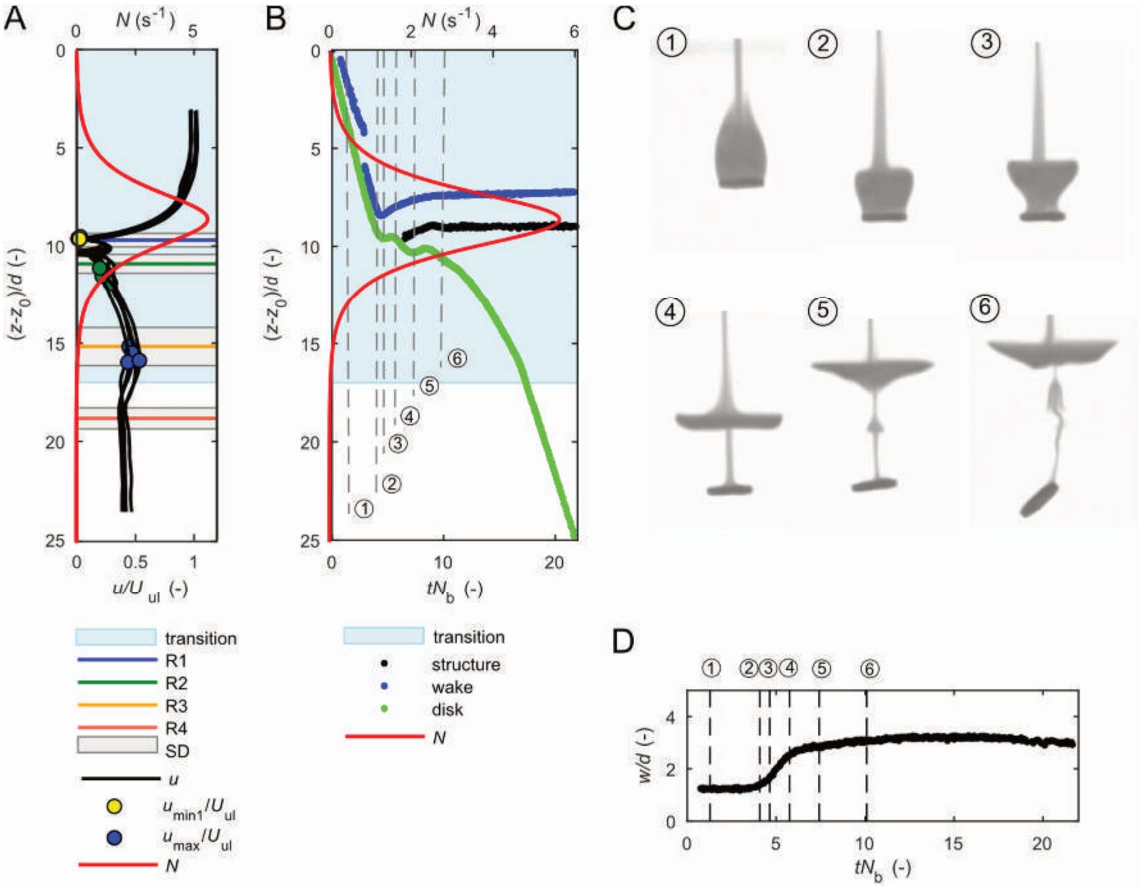

Supplementary Figure S9. Evolution of wake structure and settling dynamics of a disk for sample data SET3E1%. (A) Normalized settling velocity of disks as a function of vertical position $(z\text{-}z_0)/d$, where $z = z_0$ is the position of upper boundary of density transition. (B) Normalized position of the upper edge of a disk (green curve), lower edge of a wake (blue curve) and swelling structure on a jet (black curve) as a function of time, tN_b. Numbers indicate time instants shown in panel C. (C) Time sequence of disk translating through density transition showing selected instants; the instants are indicated in panels B and D. See supplementary video Movie 1. (D) Temporal evolution of wake's width, w. Please refer to the text for details.

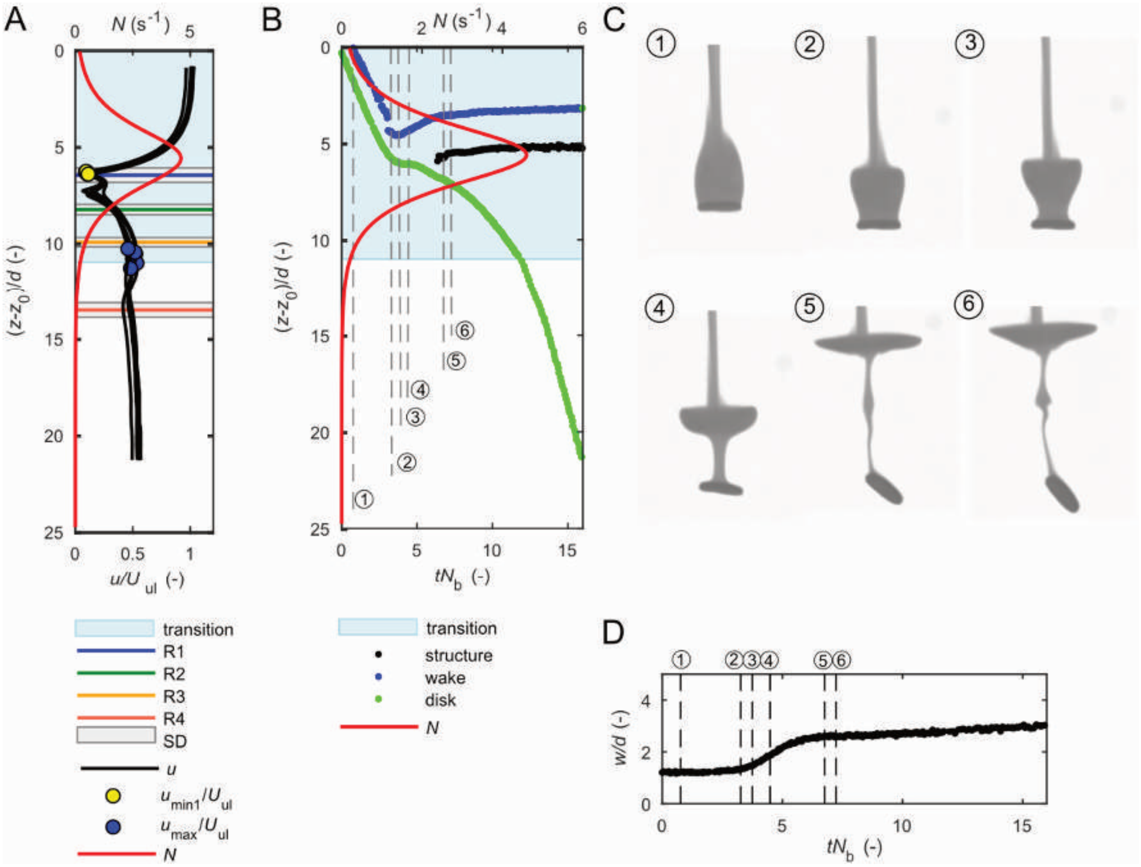

Supplementary Figure S10. Evolution of wake structure and settling dynamics of a disk for sample data SET3E2%. (A) Normalized settling velocity of disks as a function of vertical position $(z\text{-}z_0)/d$, where $z = z_0$ is the position of upper boundary of density transition. (B) Normalized position of the upper edge of a disk (green curve), lower edge of a wake (blue curve) and swelling structure on a jet (black curve) as a function of time, tN_b. Numbers indicate time instants shown in panel C. (C) Time sequence of disk translating through density transition showing selected instants; the instants are indicated in panels B and D. See supplementary video Movie 2. (D) Temporal evolution of wake's width, w. Please refer to the text for details.

Supplementary Table 1. Experimental conditions. ρ_{ll} – density of lower layer, ρ_{ul} – density of upper layer, *b* – density jump given as $(\rho_{ll} - \rho_{ul}) / \rho_{ul}$, *T* – temperature of liquid, N_b - Brunt-Vaisala buoyancy frequency evaluated from Eq. (3), N_{max} – maximum Brunt-Vaisala buoyancy frequency, U_{ul} – terminal velocity in the upper layer, SD – standard deviation, Re_{ul} – Reynolds number in the upper layer evaluated from Eq. (5), Ar_{ul} – Archimedes number in the upper layer evaluated from Eq. (7), Fr_{ul} – Froude number in the upper layer evaluated from Eq. (9), L_t – transition thickness defined as a region where $N > 0.2$ s^{-1}, *d* – disk diameter.

Experimental test	ρ_{ll} (kg m^{-3})	ρ_{ul} (kg m^{-3})	*b* (-)	*T* (°C)	N_b (s^{-1})	N_{max} (s^{-1})	U_{ul} ± SD (m s^{-1})	Re_{ul} (-)	Ar_{ul} (-)	Fr_{ul} (-)	L_t/d (-)	No. of repetitions
Set1E1%χ7	1030	1005	0.025	21.8	4.571	5.560	0.0146 ± 0.0002	43.8	15.95	1.07	10.7	5
Set1E1% χ60							0.0030 ± 0.0003	9.1	6.09	0.22		4
Set1E1% χ5							0.0125 ± 0.0001	25.0	10.63	1.37	16.0	4
Set1E1% χ40							0.0028 ± 0.0002	5.6	4.06	0.31		5
Set1E2% χ7	1031	1013	0.018	21.7	3.499	4.263	0.0129 ± 0.0004	38.7	14.06	1.23	12.3	4
Set1E2% χ60							0.0031± 0.0002	9.3	5.54	0.30		5
Set1E2% χ5							0.0112 ± 0.0001	22.4	9.37	1.60	18.5	5
Set1E2% χ40							0.0030 ± 0.00004	6.0	3.69	0.43		3
Set1E3% χ7	1031	1020	0.010	22.1	3.853	4.683	0.0113 ± 0.0003	34.0	11.83	0.98	6.0	4
Set1E3% χ60							0.0029 ± 0.0002	8.7	4.90	0.25		4
Set1E3% χ5							0.0100 ± 0.0008	20.0	7.88	1.29	9.0	5
Set1E3% χ40							0.0025 ± 0.0005	5.1	3.27	0.33		2
Set2E1% χ7	1031	1005	0.026	22.4	4.272	5.199	N/D	N/D	15.98	N/D	12.3	5
Set2E1% χ60							N/D		6.10	N/D	18.5	4
Set2E1% χ5							N/D		10.65	N/D		5
Set2E2% χ7	1032	1013	0.019	22.0	3.864	4.709	N/D	N/D	13.89	N/D	11.0	5
Set2E2% χ60							N/D		5.49	N/D	16.5	4
Set2E2% χ5							N/D		9.26	N/D		6
Set2E3% χ7	1031	1019	0.011	22.0	2.361	2.874	N/D	N/D	12.26	N/D	15.3	6
Set2E3% 60							N/D		5.02	N/D	23	3
Set2E3% χ5							N/D		8.17	N/D		6
Set3E1% χ7	1031	1005	0.026	22.9	2.218	5.598	N/D	N/D	15.93	N/D	17.0	5
Set3E2% χ7		1012	0.019	22.0	2.332	4.572	N/D	N/D	14.20	N/D	11.0	6
Set3E3% χ7		1020	0.011	21.4	1.745	3.267	N/D	N/D	11.95	N/D	11.3	6

Supplementary Table 2. Comparison between the residence time of disk in the transition layer measured in the experiments (a mean value), $t_{r\ exp}$, and evaluated from the Stokes approximation for equivalent sphere, $t_{r\ Stokes}$.

**Exp**	$t_{r\ Stokes}$ **[s]**	t_r **[s]**	$t_{r\ Stokes} / t_r$ **[-]**
Set1E1%χ7	1.1	4.7	0.23
Set1E1%χ60	2.7	42.4	0.06
Set1E1%χ5	1.9	4.9	0.39
Set1E1%χ40	4.7	27.1	0.17
Set1E2%χ7	1.5	5.6	0.27
Set1E2%χ60	3.5	36.4	0.10
Set1E2%χ5	2.5	5.7	0.44
Set1E2%χ40	6.1	30.6	0.20
Set1E3%χ7	0.9	3.0	0.30
Set1E3%χ60	2.0	22.0	0.09
Set1E3%χ5	1.5	3.2	0.47
Set1E3%χ40	3.5	19.1	0.18
Set2E1%χ7	1.3	5.8	0.22
Set2E1%χ60	3.3	42.0	0.08
Set2E1%χ5	2.3	5.8	0.40
Set2E2%χ7	1.4	5.9	0.24
Set2E2%χ60	3.3	38.0	0.09
Set2E2%χ5	2.5	5.7	0.44
Set2E3%χ7	2.0	7.3	0.27
Set2E3%χ60	4.7	35.7	0.13
Set2E3%χ5	3.4	7.8	0.44

Supplementary Movie S1 (separate file). Settling of a 3-mm-diameter disk with the evolution of a wake behind the particle, wake visualized using a dye. Sample data from SET3E1%. Settling process described in the Results section of the main text and accompanying figures presented in Supplementary Figure S8.

Supplementary Movie S2 (separate file). Settling of a 3-mm-diameter disk with the evolution of a wake behind the particle, wake visualized using a dye. Sample data from SET3E2%. Settling process described in the Results section of the main text and accompanying figures presented in Supplementary Figure S9.

Supplementary Movie S3 (separate file). Settling of a 3-mm-diameter disk with the evolution of a wake behind the particle, wake visualized using a dye. Sample data from SET3E3%. Settling process described in the Results section of the main text and accompanying figures presented in Fig. 3.
